# The Effect of Milled Municipal Solid Waste Incineration Bottom Ash on Cement Hydration and Mortar Properties

**DOI:** 10.3390/ma16062528

**Published:** 2023-03-22

**Authors:** Jurgita Malaiškienė, Edmundas Spudulis, Rimvydas Stonys

**Affiliations:** Laboratory of Composite Materials, Faculty of Civil Engineering, Institute of Building Materials, Vilnius Gediminas Technical University, 10223 Vilnius, Lithuaniarimvydas.stonys@vilniustech.lt (R.S.)

**Keywords:** municipal solid waste incineration bottom ash, cement hydration, mortar properties

## Abstract

Large amounts of municipal solid waste incineration bottom ash (MSWI BA) are formed worldwide, and this quantity is growing because of the establishment of new waste-to-energy plants. This waste is generally kept in landfills but can be used for the manufacturing of cementitious building materials. This article analyzes the use of MSWI BA as a microfiller in cement mortars. The effects of MSWI BA on the properties of cement binder and mortar were analyzed by using them separately or in combination with other microfillers: milled quartz sand, metakaolin, milled glass, and microsilica. This article investigates the flowability of cement-based mixtures, the volume change as a result of the evolution of hydrogen gas, cement hydration, XRD, TG, the physical and mechanical properties of the mortar samples, and leaching. The addition of milled MSWI BA in cement mortars was found to significantly increase slump flow; therefore, MSWI BA can be used as a microfiller. The addition of metakaolin changed the kinetics of H_2_, which evolved due to the reaction between Al and alkali, and had a positive effect on the mechanical properties of cement mortar.

## 1. Introduction

In the world, about 3.5 million tons of municipal solid wastes are generated every day [1,2]. Many countries solve the problem of such a large amount of waste by incineration. The incineration of municipal solid waste generates bottom ash (MSWI BA), fly ash (MSWI FA), and air pollution control particles trapped in the filters that protect the ambient air from pollution [3,4]. Worldwide, around 1.3 billion tons of MSWI BA were accumulated by 2012. With new waste-to-energy plants being built, this amount is expected to increase to 2.2 billion tons by 2025 [5,6]. Usually, MSWI BA is kept in landfills and is beginning to be used in road construction, but it would be important to use MSWI BA in cementitious materials, which are the most popular building materials in the world. MSWI BA is mostly composed of glass, ceramics, sand, metal, and unburnt organic matter [7,8]. Compared to MSWI FA, MSWI BA contains significantly fewer heavy metals and other toxic substances and therefore is more acceptable for reuse [9]. Analysis of the chemical and mineral composition of MSWI BA showed that this waste can be used as secondary raw material in the construction sector [10]. In the composition of MSWI BA is glass, which is finely milled and acts as a pozzolan [11]. The MSWI BA fraction of 4–16 mm means it is used mainly as coarse aggregate in concrete, while the use of finer fractions as part of cement replacement or as coarse aggregate has not been studied often [3,12]. The main disadvantages of the MSWI BA fraction being below 3 mm are: (1) high porosity (increases water demand and reduces concrete strength) [13] and (2) hydrogen gas, which evolves during the reaction of metallic aluminum particles with alkalis during the binding of cementitious materials, increasing the volume of mortar and causing the development of micro- and macrocracks in the structure [14,15,16,17,18]. This reaction of aluminum with alkaline substances follows the following equation:

nAl + X(OH)n + H_2_O → X(AlO)_2n_ + 3n/2H_2_ (1)

During cement hydration, the decrease in the volume of reaction products compared to original materials leads to the so-called autogenous shrinkage [19]. This process is particularly pronounced in high-strength, self-compacting concretes, due to the higher cement and fine aggregate content. The use of lightweight wet aggregates [20] is effective in removing such effects through the internal curing process. However, the use of milled MSWI BA as a microfiller in high-strength, self-compacting concretes can cause expansion rather than shrinkage effects, due to the presence of dispersed aluminum (Al) [21,22]. In an alkaline concrete medium, 1 g of Al causes 1.24 l of hydrogen gas to evolve under standard conditions. The aluminum content in MSWI BA stored at the Klaipeda Region Waste Management Centre (KRATC) (Lithuania) is reported to be 22 kg/t, while in Germany, it is reported to be 51 to 100 kg/t [23]. It was found [24] that the swelling of MSWI BA-modified cement samples due to the evolution of hydrogen gas is the most intense during the first 50–150 min after mortar mixing. After that, hydrogen gas evolution actually stops.

The leaching of potentially toxic elements should also be mentioned as a disadvantage of MSWI BA [25,26,27]. These include heavy metal chlorides and sulphates, most of which are found in the fraction below 0.25 mm. It is recommended to separate this fraction for the use of MSWI BA in cementitious materials [28,29]. However, the results of other researchers [30,31] on cementitious materials with MSWI BA have shown that heavy metal leaching is low and within the standard requirements, as the soluble parts of MSWI BA are bound by the hydration products of cement minerals.

In order to use MSWI BA in cementitious materials, they need to be prepared in a special method, which may include washing, heat treatment (hydrothermal cleaning, melting), stabilization by the addition of hydraulic binders, preservation in the natural environment, etc. [32]. It is recommended for MSWI BA to age first for at least 3 months under natural conditions to allow carbonization and hydration of the active phases [33,34]. Studies [6,35,36] show that after ageing in the natural environment, the pH of MSWI BA decreases from 12 to 10 and sometimes to 7, depending on the nature of the incinerated waste and storage conditions. Reburning of MSWI BA at 600–800 °C has positive effects, such as lower aluminum content, higher amounts of gehlenite and mayanite, and no swelling of the samples during molding [17,37].

The negative effects of metallic aluminum can be addressed by milling MSWI BA. It has been found that metallic aluminum particles are coalesced during the milling and can be removed by sieving through a 0.5 mm sieve. The addition of 5% milled MSWI BA (finer than 125 µm) does not significantly change the hydration behavior of cement; the total heat release is similar to that of cement, but the compressive strength of the specimens is reduced by 7% to 9% after 28 curing days [3].

The particle size was found to have a significant effect on the pozzolanic activity of MSWI BA, which can vary in the range of 250–550 mg/g, as determined by the Chapelle test [38]. The pozzolanic activity of MSWI BA depends on the glass and ceramic residue content in the ash, which may be approximately 40%. Finely milled MSWI BA (average particle size 10 µm) is reported to accelerate cement hydration and increase the compressive strength of cement specimens, especially at 90 days of curing. The compressive strength of the cement samples modified with fine MSWI BA is approximately 100 MPa, while the compressive strength of the cement samples modified with MSWI BA milled to an average particle size of 100 µm is approximately 94 MPa.

Literature analysis shows that the structure and chemical composition of MSWI BA varies over a wide range. Most of the bottom ash parameters can be stabilized by milling, and a standardized product can be produced. This product can be used to replace part of the cement in plain concretes and can be used as a microfiller for the production of self-compacting, high-strength concretes.

The aim of this research work is to investigate the influence of milled MSWI BA on cement hydration, rheological properties of cement mortars, kinetics of hydrogen evolution due to residual Al, and physical mechanical properties of hardened mortar. In order to evaluate milled MSWI BA as a microfiller, other microfillers with different properties were used to analyze the synergetic effect of MSWI BA and milled quartz sand, metakaolin, milled glass, and microsilica. Furthermore, in this work, the hydrogen evolutions of different compositions due to residual Al were analyzed.

## 2. Materials and Methods

In this work, MSWI BA from UAB Fortum Heat Lietuva, Klaipeda (Lithuania) was used. Municipal waste was incinerated in this plant at approximately 1100 °C. The MSWI BA was aged in piles under natural conditions for 6 months. The aged material was fractionated to separate the magnetic metals. The 4/16 fraction was used for further analyses and was dried at 105 °C, crushed with a jaw crusher, and milled in a ball mill.

CEM I 42.5R cement from JSC Akmenes cementas (Lithuania) was used. The following materials were used as alternative microfillers:

- Milled quartz sand (MQ), JSC Anykščių kvarcas (Lithuania). Average particle size: 8.5 µm; d_10_—0.7 µm; d_50_—5.8 µm; d_90_—20.5 µm;
- Metakaolin (ME), JSC Stikloporas (Lithuania). Average particle size: 20.4 µm; d_10_—2.6 µm; d_50_—16.8 µm; d_90_—44.5 µm.
- SiO_2_ microspheres (MS), RW Silicon GmbH (Germany). Average particle diameter: 3.2 µm; d_10_—0.1 µm; d_50_—0.6 µm; d_90_—10.3 µm.
- Milled glass (MG), JSC Stikloporas (Lithuania). Average particle diameter 15.1 µm; d_10_—2.7 µm; d_50_—13.9 µm; d_90_—29.2 µm.

The particle size distribution of the materials was investigated using laser diffraction (Cilas 1090, Germany).

The fine aggregate used in the mortars was quartz sand (QS) from JSC Anykščių kvarcas (Lithuania), which had a fraction of 0.5–1 mm.

A polycarboxylate-based superplasticizer (SP) Glenium ACE 430 (FM) was used to study the influence of microfillers on the rheological properties of mortars.

The chemical compositions of the materials used are given in Table 1. Chemical analysis of the tested materials was carried out using X-ray fluorescence (Rigaku ZSX Primus IV, Japan). The following spectrometer parameters were used: Rh anode, 4 kW, 60 kV, sample diameter 40 mm, and height 3 mm. The powdered materials were compressed with a force of 200 kN.

In addition, the following properties of the milled MSWI BA were determined: the weight of residue on a 90 µm sieve—4%; bulk density—0.91 g/cm^3^; particle density—2.73 g/cm^3^; specific surface area (Blaine method)—491.0 m^2^/g; pH (suspension concentration 20%)—9.5; and electrical conductivity (suspension concentration 20%, after 3 h)—3.65 S/m.

**Figure 1:**
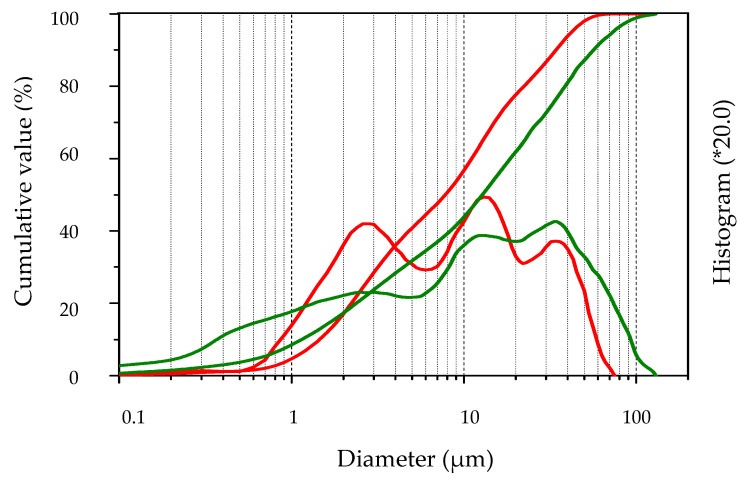
Particle size distribution curves of cement (green) and milled MSWI BA (red).

The particle size distributions of the milled MSWI BA and cement are shown in Figure 1. The curves are quite similar; the average diameter of the milled MSWI BA particles was approximately 13.7 µm (d_10_—1.6 µm; d_50_—9.2 µm; d_90_ –34.7 µm) and 15.6 µm (d_10_—0.8 µm; d_50_ –6.7 µm; d_90_—46.4 µm) for cement particles.

**Figure 2:**
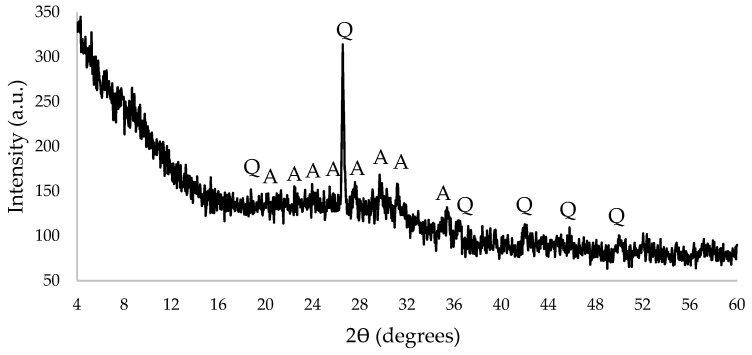
X-ray of MSWI BA (Q—quartz, A—anorthite).

The X-ray of MSWI BA is shown in Figure 2. Qualitative phase analysis of MSWI BA was performed by X-ray diffraction (DRON-7, Russia). A graphite monochromator was used to obtain the X-ray Cu Kα spectrum (λ = 0.15418 nm). The main minerals contained in MSWI BA were found to be quartz and anorthite; the hill at 20–33° also indicated the presence of amorphous SiO_2_.

The heat flow and the total heat release rate during the hydration of the solvent (distilled water) were measured with the microcalorimeter Tam Air III (TA Instruments, New Castle, DE, USA). Glass ampoules with 20 mL of capacity were used for the experiments. They were filled with 3 g of dry materials and placed in the microcalorimeter. After reaching a constant temperature of 25 ± 0.1 °C, 1.5 g of distilled water was added to the ampoule. The resulting suspension was stirred at 2–3 rpm for 20 s. Heat flow was measured for 40 h.

Changes in cement mortar volume during curing were determined by continuous hydrostatic weighing [39]. The mortar sample in a latex container was placed in the container with silicone grease and weighed every minute for a period of 24 h. Silicone grease (Molyduval Silo D350, density: 970 kg/m^3^) helped prevent water migration through the latex membrane due to osmotic effects. The measurement of the change in mortar volume was started 10 min after contact between water and dry materials. During the test, some of the evolved gas collected above the mortar and was included in the total volume of the sample.

The rheological properties of the cement mortars were tested using a cone with a diameter of 3 cm and a height of 5 cm, according to LST EN 12706. The slump flow measurement was started 5 min after mixing the mortars. The slump was measured every 5–10 min for 90 min.

The compositions of the cement mortar are given in Table 2. In order to minimize the formation of cracks due to H_2_ evolution, the maximum content of MSWI BA was upped to 10%. In the mixtures with alternative microfillers, a part of the MSWI BA was replaced by 2.5% of the other microfillers. The superplasticizer content was kept constant in all mixtures at 1.8% (more than 100%), with a W/S (water/solid ratio) of 0.12.

Cement pastes (Table 3) (compositions without QS) were used to study the change in volume of the samples due to the evolution of the hydrogen gas and cement hydration properties. The superplasticizer content was kept constant in all mixtures: 1.3% (more than 100%), with a W/S of 0.24. In these compositions, W/S was higher compared to M1–M9; In Table 3, compositions of cement pastes without the fine QS aggregate are presented.

The procedure for the preparation of the samples was as follows: cement, MSWI BA, alternative microfillers, and sand, if needed, were mixed dry in a Hobart mixer according to EN 196-1 at the first speed for 1.5 min (140 ± 5 min^−1^); then, water and superplasticizer were added; the mixture was mixed for 1.5 min at the first speed; the mixer was stopped for 30 s; afterwards the mixing continued at the second speed (285 ± 10 min^−1^) for 1 min. After mixing, 160 × 40 × 40 mm samples were molded. After 24 h, the samples were demolded and stored in a chamber at a 20 ± 2 °C temperature and 65% humidity for the curing time required for the tests (28 days).

The densities of the samples cured for 28 days (according to LST EN 1015-11) were calculated based on their mass and volume; three samples of each composition were tested. The flexural (3 samples of each composition) and compressive strengths (6 samples of each composition) of the samples were determined after 28 days of curing with the Tinius Olsen H200 KU press, according to the requirements of LST EN 196.

To determine the leaching of harmful substances, the samples were prepared according to LST EN 12457-2 in a 10 L/kg water–solid ratio, with a particle size below 4 mm. The samples were tested after 28 days of curing. The electrical conductivity and pH of the filtered eluate were measured after the agitation of the leachate for 24 h. The chemical composition of the eluate was analyzed using a Rigaku ZSX Primus IV XRF spectrometer.

## 3. Results

### 3.1. Tests on Cement Mortar and Hardened Cement Paste

**Figure 3:**
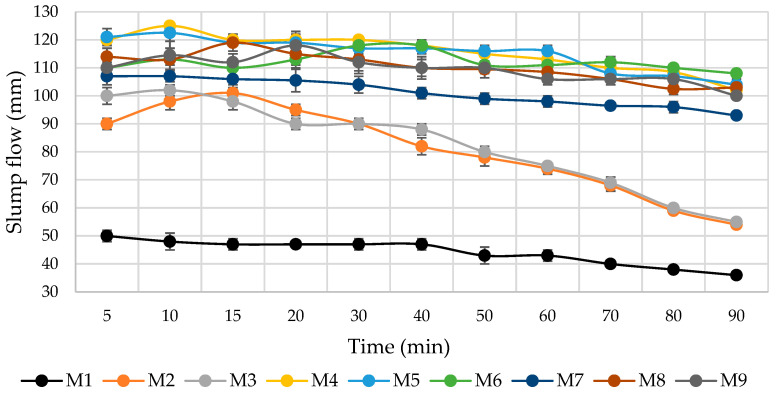
Slump flows of cement mortars.

The cement mortar slump flow tests showed (Figure 3) that milled MSWI BA could be used as an additional microfiller. A total of 10% of MSWI BA (M5) was effective at increasing the slump flow of the mortar with little variation over a 90 min period. Compared to the control specimen M1, where no MSWI BA was used, the slump flow increased from 50 mm to 90–120 mm, depending on the composition. (Higher slump values were obtained for compositions with 7.5% and 10.0% of MSWI BA in compositions M5–M9.) In other scientific works [2], it was found that the slump flow depended on the size of MSWI BA: a high workability value was established for mixtures with fine MSWI BA, and this was attributed to the loss of cohesion between aggregates and cement paste; if MSWI BA was used as the coarse aggregate, the slump flow usually decreased, due to the high water absorption of MSWI BA, compared to natural aggregate. It could be that in our tests, 5% of the MSWI BA used was too small of a quantity to influence the cohesion (slump flow after 30 min decreases), but a higher amount (10%) could have a significant impact and increase slump flow. However, water separation was observed in mixtures where only MSWI BA (M3, M5) was used. Water separation was not observed in the compositions with the addition of milled quartz sand (M4, M6), metakaolin (M7), milled glass (M8), and microsilica (M9), due to the high surface areas of these additives. A high percentage of fines in milled sand can affect the water demand required to obtain constant workability; additionally, the produced sand typically has more angular particles, which also tend to increase the water demand, as they affect the content of the voids and frictional properties in concrete [40,41,42].

**Figure 4:**
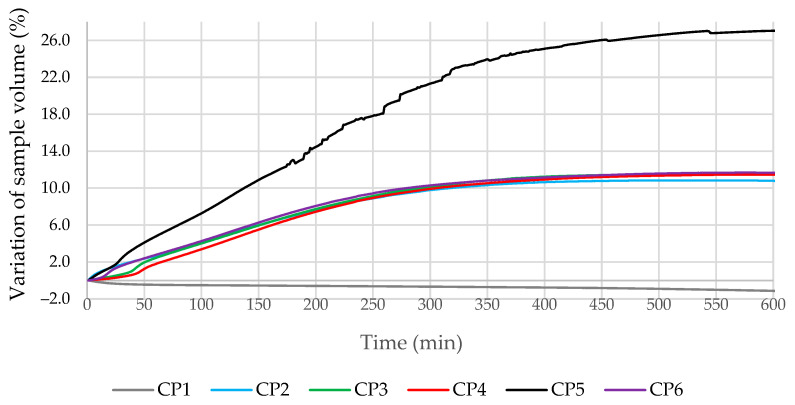
Test results for the changes in cement paste volumes due to hydrogen gas evolution.

Cement pastes without quartz sand were used to study the change in the volume of the samples as a result of the evolution of the hydrogen gas. During the tests, some of the H_2_ gas evolved without affecting the volume of the sample (above the cement paste) but were included in the overall change in volume. The compositions of the mixtures tested are given in Table 3, and the data obtained are shown in Figure 4. The addition of milled glass was found to significantly increase the evolution of the gas (about 25%, compared to the CP1 control specimen CP1). When milled MSWI BA was used without additional additives or the addition of milled quartz sand, microsilica, or metakaolin, the changes in the volume of the samples were similar, with an increase of 11–12% after 10 h. The most intense changes in the volume of cement paste occurred in the first 300 min after mixing. Meanwhile, the volumes of the cement pastes with SP (without microfillers) were reduced by approximately 1% after 10 h. P. Paulini [43] used a similar method for the evaluation of cement shrinkage and established that such a method was appropriate to analyze the efficiency of additives in cement paste, especially for the initial reaction period, and determined that the volume of cement samples without special additives decreased.

**Figure 5:**
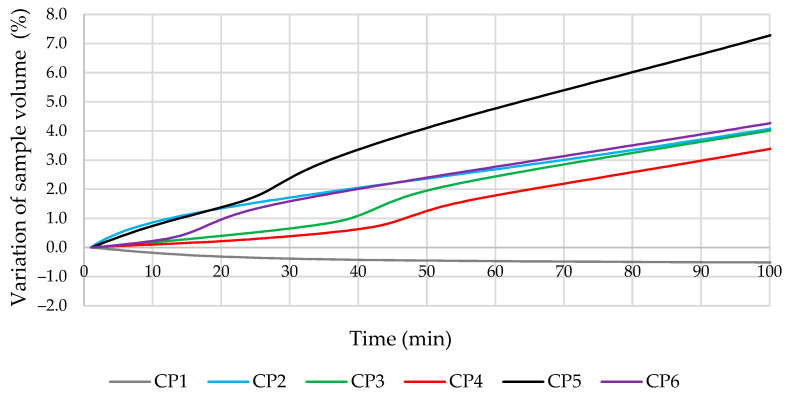
Test results for the change in specimen volumes due to hydrogen gas evolution.

The effect of the additives used on the change in cement paste volume during the first 100 min is shown in detail in Figure 5. The volume of additive-free cement mortar (CP1) decreased in the first minutes of the test. A certain induction period was observed in all mortars containing other microfillers. At the initial stage, the gas evolution in the modified specimens was slower compared to mortar CP2. This effect was observed even with the addition of milled glass microfillers (CP5). Apparently, these additives influenced the variation in pH of the liquid phase of cement mortars, especially MG, due to the large amount of Na in their chemical composition. The pH in the composition with MG was higher compared to other compositions, in which a large amount of SiO_2_ was used and the pH was lower. The most effective way to reduce the evolution of the gas at the beginning of the process was by adding 2.5% metakaolin (CP4). In literature [44], it was established that the addition of metakaolin reduces pH, due to the pozzolanic reaction that occurs in cementitious materials. In an environment with less pH (less alkaline), reaction according to the Equation (1) is not so intensive.

The densities of the hardened cement mortar samples M1–M9 were found to vary from 2200 kg/m^3^ to 2300 kg/m^3^. The lowest densities were found in samples with the highest MSWI BA contents and without additional additives, due to their lower densities compared to cement and a higher evolution of the H_2_ gas. Similar trends have been presented in other works [45,46], where the densities decrease with increasing amounts of MSWI BA.

**Figure 6:**
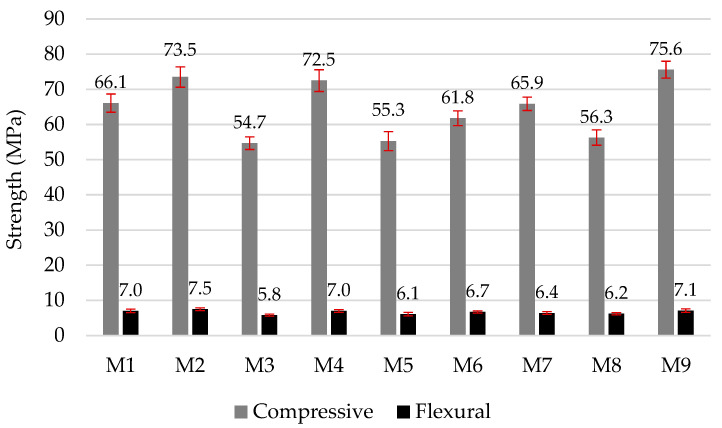
Compressive and flexural strengths of mortars at 28 days.

In literature [2], it is written that the use of MSWI BA in cement mortar and concrete causes a reduction in compressive, tensile, and flexural strength, due to the cement dilution effect, and a higher evolution of the H_2_ gas, due to metal Al; however, this can be alleviated by limiting the level of the MSWI BA replacement. In this work it was found that the flexural strength varied from 5.8 MPa to 7.5 MPa, and the compressive strength varied from 54.7 MPa to 75.6 MPa (Figure 6). The highest strength values were obtained for samples in which 2.5% of microsilica (M9) was used together with milled MSWI BA; these results can be explained by the high reactivity of microsilica and the greater amount of calcium hydrosilicates formed (CSH) [47]. The composition M7 modified with the 2.5% ME additive also showed relatively high strength, due to the pozzolanic activity of metakaolin. It was also found in literature [48] that the basic physical properties of metakaolin were very similar to PC. The compressive strength of sample M7 with metakaolin was similar to that of the control sample M1, although M7 contained 10% less cement. A comparison of the M6–M9 compositions, which contained the same amount (7.5%) of MSWI BA and 2.5% of other pozzolanic additives, showed that samples with microsilica had the highest compressive strength, due to the highest pozzolanic activity and the largest surface area of microsilica, followed by samples containing metakaolin, while samples with milled glass had the lowest strength. The test data showed that the addition of milled MSWI BA (up to 33% by weight of cement) reduced the compressive strength of the specimens by 10%. The mechanical properties of the samples were adversely affected by the addition of milled glass (MG), which intensified the evolution of H_2_, due to the highest alkalinity. In contrast, the addition of metakaolin, which reduced the alkalinity of the environment, inhibited these processes. On the basis of the analysis of the change in sample volume due to the aluminum and alkali reactions and the mechanical properties of the samples, the metakaolin additive was chosen for further investigations.

### 3.2. Tests on Cement Hydration

**Figure 7:**
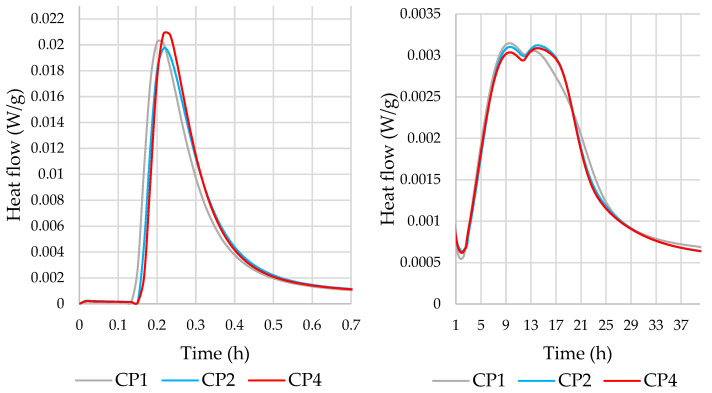
Effect of cement paste composition on the heat flow rate.

**Figure 8:**
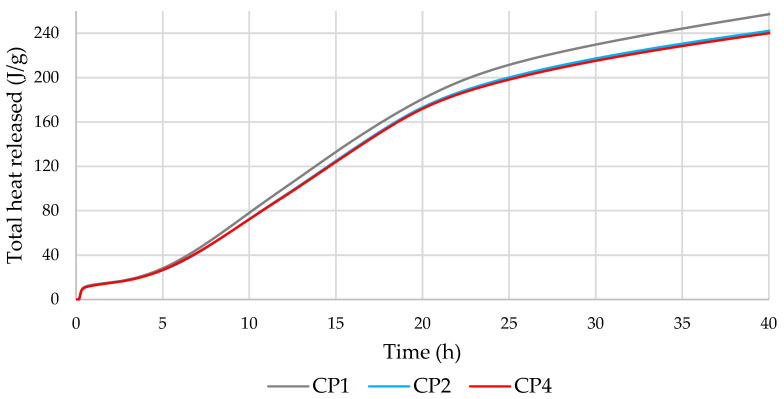
Effect of cement paste composition on the total heat released.

The compositions used for the microcalorimetry are shown in Table 3. After adjusting the results to the same cement content, the microcalorimetric curves (Figure 7 and Figure 8) showed that the heat flow rate and the total amount of heat released were very similar and overlapped for all mixtures. The additives used, including MSWI BA, did not have a significant effect on the amount of heat released and the kinetics of cement hydration. Previous literature [49,50] found that the addition of MSWI BA provided effective nucleation sites for CSH to form and therefore increased the heat output rate during the acceleratory period, despite a 20% cement dilution.

The results of the quantitative analysis of the mineral phases by Rietveld XRD showed that the replacement of 9.5%–12.5% of cement with finely dispersed MSWI BA caused the contents of portlandite and ettringite to decrease and the amount of the amorphous phase to increase (Table 4). The use of 12.5% MSWI BA (CP2) reduced the portlandite content by approximately 25% after 7 days of curing and by approximately 3% after 28 days of curing. The amorphous phase increased by about 35% and 3%, respectively. Therefore, the effect of MSWI BA was stronger during the first days of curing, with results similar to those of the control samples. The most significant effect on phase changes was observed after 28 days of curing when 12.5% of the cement was replaced with 9.5% MSWI BA and 3% metakaolin. The portlandite content decreased by approximately 20%, the content of ettringite decreased by approximately 30%, and the amorphous phases increased by approximately 10%. These results lead to the conclusion that MSWI BA promotes the formation of a higher amount of amorphous phase, and thus, the hardened cement paste should have a denser structure and higher strength.

**Figure 9:**
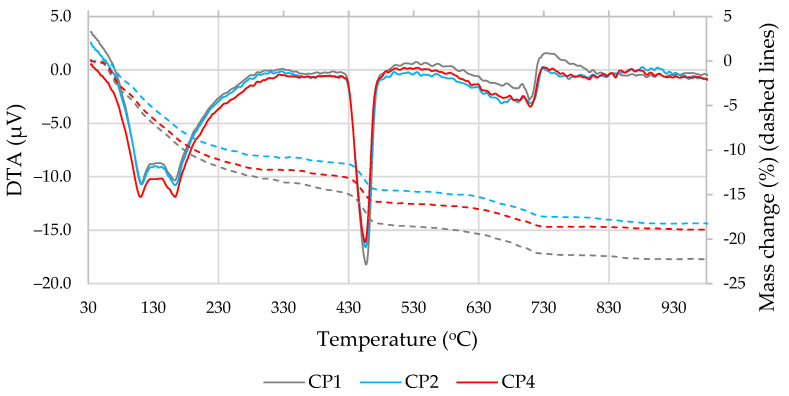
DTA and TG results after 7 days of curing.

**Figure 10:**
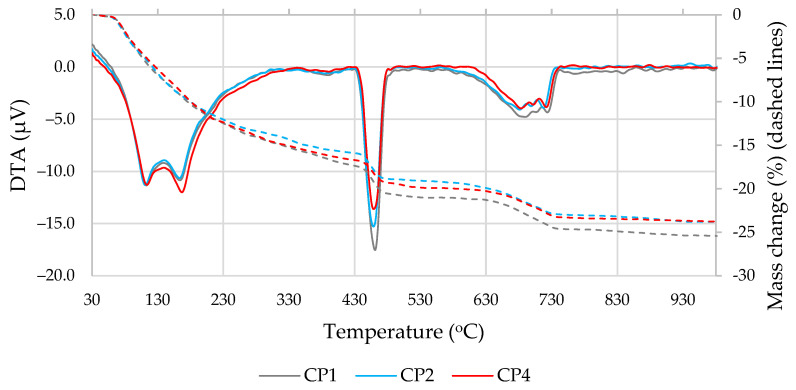
DTA and TG results after 28 days of curing.

The results of the thermal analysis (Figure 9 and Figure 10 and Table 5) showed that the decomposition of the hydration products in all compositions took place at the same temperature; the mass loss at temperatures up to 400 °C was similar in all compositions, with greater differences recorded at 430–560 °C (portlandite decomposition) and 600–750 °C (carbonate decomposition) [51]. As portlandite is one of the most important hydration products of Portland cement, its content was examined in more detail (Table 5). Naturally, the portlandite content decreased when part of the cement was replaced with additives; the most significant decreases of approximately 22% and 12% were observed at 7 and 28 days, respectively, in the samples where the MSWI BA and ME additives were used. The portlandite content also decreased in the specimens modified with MSWI BA only. To ensure that the decrease in portlandite content was not only due to the cement dilution effect, its content was adjusted to the same amount of cement (Table 5). It was found that in the samples with MSWI BA, the portlandite content also decreased with the same amount of cement. This decrease in portlandite can be explained by the pozzolanic effect of MSWI BA. The carbonate content in the samples with cement and with MSWI BA-modified cement was found to be similar, but the addition of ME resulted in a lower carbonate content at both 7 and 28 days.

### 3.3. Testing the Leaching of Harmful Substances

The analysis of the substances from the crushed samples dissolved in water did not reveal the presence of any heavy metals or other particularly harmful substances in the eluate (Table 6). Leaching of certain amounts of calcium and potassium increased the pH and electrical conductivity of the eluate.

## 4. Conclusions

Milled MSWI BA was found to cause cement paste samples to swell, due to the reaction of residual Al with the alkalis released during cement hydration, which is most pronounced during the first 300 min. The addition of metakaolin reduced the evolution of H_2_ and had a positive effect on the mechanical properties of cement mortar. The induction period, which was observed during the initial stage of H_2_ evolution, was influenced by the properties of the microfiller used.

The addition of milled MSWI BA to cement mortars with superplasticizers increased the slump flow; therefore, milled MSWI BA can be used as a microfiller. Milled MSWI BA is more effective when used in combination with metakaolin.

The densities of the hardened cement mortars tested showed little variation, ranging from 2200 to 2300 kg/m^3^. The highest compressive strength values were obtained in samples modified with MSWI BA and microsilica. However, for economic reasons and smaller changes in the volumes of samples, the addition of waste metakaolin was recommended. In that case, with 10% cement replaced with 7.5% of MSWI BA and 2.5% metakaolin, the compressive strength at 28 days was similar to the strength of the unmodified samples.

The results of the microcalorimetry showed that the replacement of part (up to 12.5%) of the cement with MSWI BA did not have a significant effect on the hydration kinetics of the cement.

The most significant effect on changes in mineral phases in cement mortars was caused by the addition of a complex additive, where 12.5% of cement was replaced by 9.5% MSWI BA and 3% metakaolin. It caused an approximately 20% decrease in portlandite, 30% decrease in ettringite, and 10% increase in the amorphous phase, compared to the control sample. According to the thermal analysis results, MSWI BA and metakaolin reduced the portlandite content, and the combined effect of these additives was the most effective. These results suggest that MSWI BA also acts as a pozzolanic additive.

The leaching test for harmful substances showed that the leaching of calcium and potassium from the cement mortar samples caused the pH and electrical conductivity of the eluate to increase. Heavy metals were not detected in the leachate.

## Figures and Tables

**Table 1:** Chemical compositions of materials (%).

Material	SiO_2_	CaO	Al_2_O_3_	Fe_2_O_3_	Na_2_O	MgO	SO_3_	K_2_O	TiO_2_	Cl	ZnO	BaO	P_2_O_5_
Cement	20.0	63.4	4.57	3.46	0.14	3.71	2.73	1.30	0.32	–	0.01	0.06	–
MQ	99.2	0.07	0.57	0.05	0.03	0.02	0.02	0.02	0.05	–	–	–	–
ME	52.4	1.27	39.8	1.03	3.39	0.37	0.08	0.93	0.54	0.01	–	–	0.12
MS	96.1	0.25	0.20	0.05	0.10	0.40	0.35	1.20	–	–	–	–	–
MG	72.3	9.65	1.01	0.17	12.7	3.49	0.28	0.33	0.07	0.02	–	–	–
MSWI BA	55.6	14.5	8.3	7.4	5.8	2.3	1.1	1.6	0.7	0.15	0.3	0.2	–

**Table 2:** Compositions of the cement mortar (%) by mass.

Mark	Cement	MSWI BA	MQ	ME	MG	MS	QS
M1	35						65
M2	35		5				60
M3	35	5					60
M4	35	2.5	2.5				60
M5	30	10					60
M6	30	7.5	2.5				60
M7	30	7.5		2.5			60
M8	30	7.5			2.5		60
M9	30	7.5				2.5	60

**Table 3:** Compositions of cement pastes (%) by mass.

Mark	Cement	MSWI BA (FP)	MQ	ME	MG	MS
CP1	100	0	0	0	0	0
CP2	87.5	12.5	0	0	0	0
CP3	87.5	9.5	3	0	0	0
CP4	87.5	9.5	0	3	0	0
CP5	87.5	9.5	0	0	3	0
CP6	87.5	9.5	0	0	0	3

**Table 4:** XRD Rietveld test results.

Mineral (%)	CP1	CP2	CP4	CP1	CP2	CP4
	After 7 Days			After 28 Days		
Belite	16.5	10.2	11.4	8.6	7.3	8.6
Calcium aluminum oxide	2.2	1.7	2.2	0.6	1.1	0.7
Periclase	1.2	0.8	0.6	0.1	0.0	0.1
Alite	8.5	7.6	7.0	6.5	7.7	6.0
Calcite	6.2	5.7	6.7	4.2	4.0	4.0
Portlandite	14.3	10.8	12.0	11.2	10.9	9.1
Brownmillerite	2.5	2.6	2.0	3.4	2.5	3.0
Gypsum	1.6	1.6	2.0	2.3	1.9	2.5
Ettringite	5.6	3.9	5.3	5.8	5.0	4.0
Zincite	10.0	10.0	10.0	10.0	10.0	10.0
Quartz		2.6	0.6		0.7	0.3
Amorphous phase	31.6	42.5	40.4	47.5	49.1	51.9

**Table 5:** Mass loss results for the relevant temperature ranges.

Mark	430–560 °C, %	Amount of Dry-Basis Portlandite, %	Amount of Cement-Base Portlandite, %	600–750 °C, %
**At 7 days**				
CP1	3.7	18.8	18.8	2.6
CP2	3.1	15.0	16.7	2.5
CP4	3.0	14.7	16.4	2.3
**At 28 days**				
CP1	3.6	18.8	18.8	3.4
CP2	3.3	16.9	18.7	3.5
CP4	3.2	16.5	18.3	3.2

**Table 6:** Amounts of oxides leached from the samples and properties of leachate (DW—distilled water).

Mark	P_2_O_5_ (mg/L)	CaO (mg/L)	MgO (mg/L)	Al_2_O_3_ (mg/L)	SO_3_ (mg/L)	K_2_O (mg/L)	Fe_2_O_3_ (mg/L)	Conductivity (mS)	pH	T (°C)
DW	6	4	0	0	0	0	0	0.03	6.46	22.9
M1	9	43	34	13	4	17	2	5.46	12.37	21.4
M5	8	29	27	8	3	17	0	5.16	12.33	21.2
M7	10	31	0	7	0	14	0	3.96	12.27	20.5
M8	9	28	24	7	0	14	0	5.85	12.30	21.3
M9	8	23	26	6	0	12	0	4.18	12.22	21.0

## Data Availability

Not applicable.
